# HER2 Low, Ultra-low, and Novel Complementary Biomarkers: Expanding the Spectrum of HER2 Positivity in Breast Cancer

**DOI:** 10.3389/fmolb.2022.834651

**Published:** 2022-03-15

**Authors:** Konstantinos Venetis, Edoardo Crimini, Elham Sajjadi, Chiara Corti, Elena Guerini-Rocco, Giuseppe Viale, Giuseppe Curigliano, Carmen Criscitiello, Nicola Fusco

**Affiliations:** ^1^ Division of Pathology, IEO, European Institute of Oncology IRCCS, Milan, Italy; ^2^ Department of Oncology and Hemato-Oncology, University of Milan, Milan, Italy; ^3^ Division of Early Drug Development for Innovative Therapy, IEO, European Institute of Oncology, IRCCS, Milan, Italy

**Keywords:** breast cancer, biomarkers, HER2 low expression, HER2 ultra low, targeted therapy, antibody-drug conjugate, immunohistochemistry, fluorescence *in situ* hybrdization

## Abstract

HER2 status in breast cancer is assessed to select patients eligible for targeted therapy with anti-HER2 therapies. According to the American Society of Clinical Oncology (ASCO) and College of American Pathologists (CAP), the HER2 test positivity is defined by protein overexpression (score 3+) at immunohistochemistry (IHC) and/or gene amplification at *in situ* hybridization (ISH). The introduction of novel anti-HER2 compounds, however, is changing this paradigm because some breast cancers with lower levels of protein expression (i.e. score 1+/2+ with no gene amplification) benefited from HER2 antibody-drug conjugates (ADC). Recently, a potential for HER2 targeting in HER2 “ultra-low” (i.e. score 0 with incomplete and faint staining in ≤10% of tumor cells) and MutL-deficient estrogen receptor (estrogen receptor)-positive/HER2-negative breast cancers has been highlighted. All these novel findings are transforming the traditional dichotomy of HER2 status and have dramatically raised the expectations in this field. Still, a more aware HER2 status assessment coupled with the comprehensive characterization of the clinical and molecular features of these tumors is required. Here, we seek to provide an overview of the current state of HER2 targeting in breast cancers beyond the canonical HER2 positivity and to discuss the practical implications for pathologists and oncologists.

## Introduction

Breast cancer is the most frequently diagnosed cancer in women and a leading cause of death worldwide (Sung et al., 2021). This malignancy is extremely heterogeneous in terms of clinicopathological and molecular characteristics, prognosis, and response to therapy (De Mattos-Arruda et al., 2018; Lopez et al., 2019; Filho et al., 2021). In 15–20% of cases, breast cancers show the overexpression of HER2, usually due to gene amplification (Seshadri et al., 1993). Given that HER2 is a potent (proto)oncogene, these tumors harbor a more aggressive behavior compared to HER2-negative breast cancers (Hamilton et al., 2021). The introduction of anti-HER2 monoclonal antibodies (e.g., trastuzumab, pertuzumab) back in the 90 s revolutionized the treatment landscape of HER2+ breast cancer, drastically improving the life expectations of these patients (Dieci and Miglietta 2021). Since then, several studies and harmonization efforts have been carried out to improve the sensitivity and specificity of HER2 pathological assessment, which is now considered highly reliable (Viale and Munzone 2019). However, the introduction of novel anti-HER2 antibody-drug conjugates (ADC) strategies is questioning the existing paradigm of HER2 testing (Banerji et al., 2019; Modi et al., 2020; Corti et al., 2021a).

According to the 2018 American Society of Clinical Oncology (ASCO)/College of American Pathologists (CAP) guidelines, breast cancer is classified as HER2-positive when HER2 expression is scored as 3+ by immunohistochemistry (IHC) or 2+ IHC with gene amplification by reflex *in-situ* hybridization (ISH) (Wolff et al., 2018). For these tumors, there is a clinical recommendation for anti-HER2 targeted agents. On the contrary, tumors with IHC scores 0 and 1+, or 2+ with a negative ISH, are clinically HER2-negative because they lack a significant response to traditional anti-HER2 drugs (Pondé et al., 2018; Wolff et al., 2018). Lately, tumors with low levels of HER2 expression (i.e. IHC 1+ or 2+ with negative ISH), also referred to as HER2 “low” breast cancers, have been shown impressive response rates and progression-free survival (PFS) after ADC-based treatments (Iwata et al., 2018; Banerji et al., 2019; Schettini et al., 2021). Another rising layer of complexity is represented by the possible clinical relevance of the incomplete and faint staining in ≤10% of tumor cells displayed by a subset of IHC score 0 breast cancers. This HER2 “ultra-low” phenotype might explain some promising evidence on treatment response in HER2-negative breast cancer (Denkert et al., 2021). Recent preclinical studies on HER2 targeting in MutL-deficient estrogen receptor (ER)+/HER2-negative breast cancers have shown positive results (Punturi et al., 2021). These data might provide a further rationale for expanding our pathological armamentarium towards mismatch repair (MMR)-related biomarkers (Venetis et al., 2020b; Sajjadi et al., 2021b).

In this review, we present a thorough overview of the state of the art in breast cancer predictive pathology for HER2 targeting within and beyond the HER2 positivity spectrum. Practical technical hindrances for an accurate patient selection will be discussed in light of the most recent clinical trials.

## Rebooting HER2 Testing in Breast Cancer

### HER2-Low Status Assessment in Breast Cancer: Opportunities and Challenges

**FIGURE 1:**
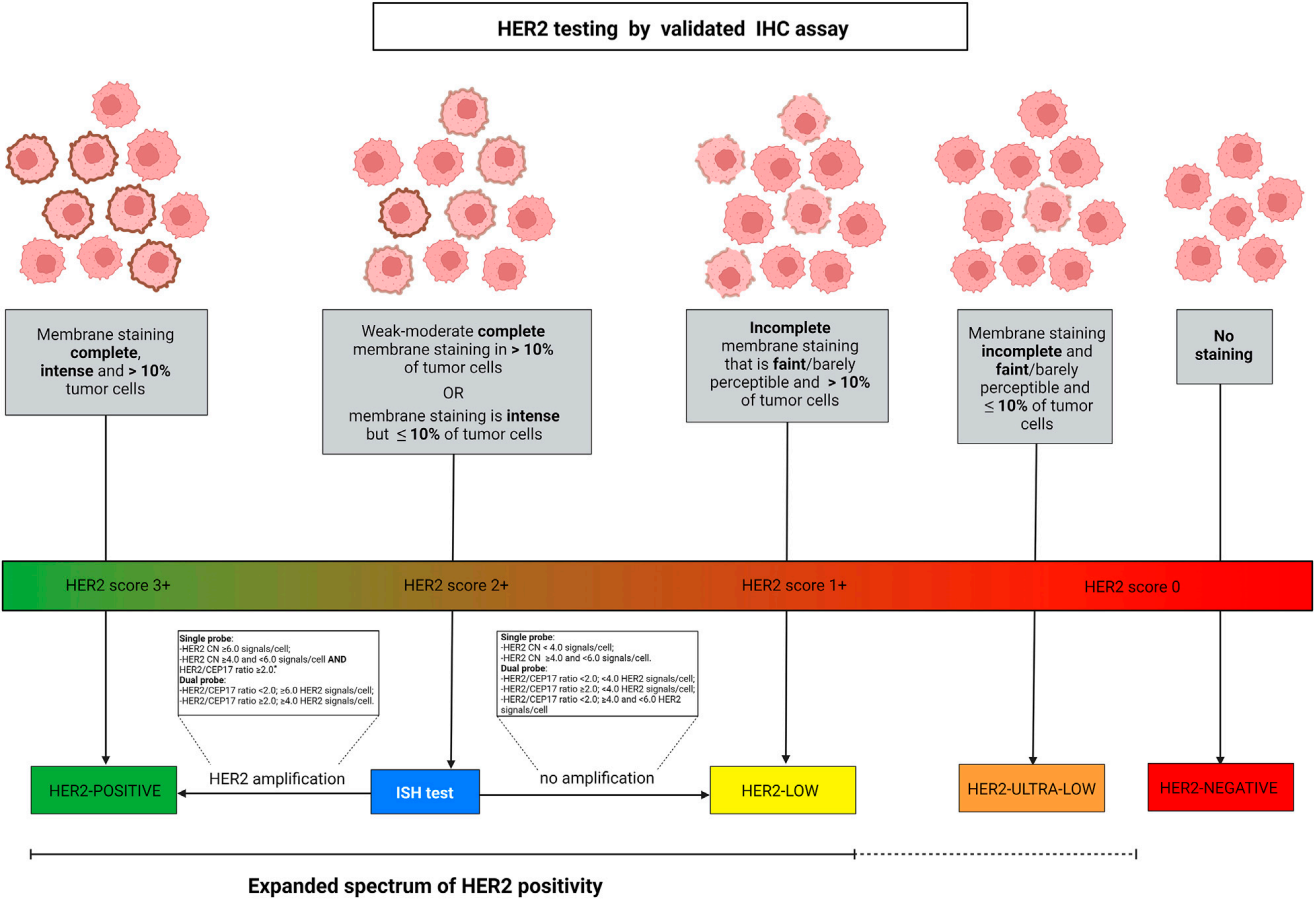
Algorithm for defining HER2 spectrum of expression according to ASCO/CAP guidelines. HER2-low diseases are identified by IHC score 2+ with negative ISH or IHC score 1+. Breast cancer is scored 2+ in case of weak-moderate complete membrane staining in >10% of tumor cells or if the membrane staining is intense but in ≤10% of tumor cells. Score 1+ is defined by faint or barely perceptible incomplete membrane staining in >10% of tumor cells. Gene amplification by ISH is assessed as detailed in the text boxes, according to the types of probes employed. Among the IHC score 0 category, two different types of expression are present, namely a complete lack of expression and the faint or barely perceptible incomplete membrane staining in ≤10% of tumor cells. Albeit HER2-negative, this latter type of tumor indeed shows the expression of the protein and can be described as HER2 “ultra-low”. The continuous versus the discontinued line depicts the different levels of evidence in the current clinical practice. Abbreviations, IHC, immunohistochemistry; ISH, *in situ* hybridization; HER2, human epidermal growth factor receptor 2. *, dual-probe ISH should be performed for final result. Source: Breast Biomarker Reporting, CAP Cancer Protocol Templates, v1.4.1.1, November 2021 update, available at: https://documents.cap.org/protocols/Breast.Bmk_1.4.1.1.REL_CAPCP.pdf. Created with biorender.com.

The discovery of HER2 in the 1980s allowed the development of therapeutical strategies that have dramatically changed the natural history of HER2+ breast cancer, with significantly improved outcomes (Coussens et al., 1985; Slamon and Pegram 2001). HER2 testing is routinely recommended for all newly diagnosed breast cancers, and a re-characterization can be performed in some cases after neoadjuvant treatment and/or in case of tumor progression if a tissue sample is available (Viale and Fusco 2021; Wolff et al., 2018). This test relies on the combination of IHC and ISH (Schalper et al., 2014). In particular, IHC is an essay that identifies and describes the HER2 protein expression pattern and intensity on the cell membrane of breast cancer cells, while ISH detects the presence of gene amplification using HER2 and CEP17 probes (Pauletti et al., 1996; Slamon et al., 1989). The first step of the HER2 testing workflow requires the performance of IHC. Based on the completeness, intensity, and percentage of cells in which the staining is identified, HER2 IHC is scored using a three-tiered system, from 0 to 3+ **(** Figure 1) (Wolff et al., 2018). In the case of an equivocal result (score 2+), ISH is used as a reflex test (Press et al., 2002). Specifically, when ISH shows an average HER2 copy number ≥6.0 signals/cell, the case is HER2+ (Wolff et al., 2018). Taken together, HER2+ breast cancer is defined by a 3+ IHC score or 2+ IHC and positivity of ISH, while tumors with HER2 IHC scores of 1+ or 2+ without ISH amplification are defined as HER2-low (Tarantino et al., 2020). More recently, beyond the striking and established efficacy of HER2-directed therapies in HER2+ breast cancer, the possibility of targeting HER2 has been explored also in HER2-low breast cancer (Fusco et al., 2013; Fusco and Bosari 2016; Meric-Bernstam et al., 2019; Kreutzfeldt et al., 2020; Sartore-Bianchi et al., 2020; Shitara et al., 2020; Siena et al., 2021). Of note, even if the HER2 pathway activation is lower in HER2-low than in HER2+ breast cancer, the new anti-HER2 ADCs allow its targeting. New ADCs contain a strong chemotherapeutic payload that is guided against tumor cells thanks to their, even low, HER2 positivity.

Although the ASCO/CAP guidelines have clearly defined the assessment criteria for HER2 status in breast cancer, HER2-low expression has not been formally defined (Tarantino et al., 2020). The precise identification of these patients is intrinsically dependent on the testing strategy and technique (Ercoli et al., 2017). The reproducibility of these tests might be affected by several pre-analytical and analytical issues (Fusco et al., 2021). Formalin fixation and artifacts, technical and biological heterogeneity represent factors that remarkably affect the analytical reliability of IHC, complicating the identification of HER2-low expression both in terms of both false positive and false negative results (Bussolati et al., 2015). These observations, along with the pooling of HER2 scores 0 and 1+ under the broader definition of HER2-negative breast cancer, may explain to a certain extent some of the discrepancies that have been observed in HER2 testing. In this context, several studies that assessed the reproducibility of HER2 testing between local and central laboratories revealed a remarkable inter-observer, intratumoral, and temporal heterogeneity of HER2 low status (Miglietta et al., 2021). Therefore, a precise assessment of HER2-low cases demands the harmonization of all methodologies coupled with straightforward and well-defined guidelines (Angerilli et al., 2021). In this regard, the role of pathologists is crucial, thus specific training and particular attention during HER2 testing is warranted. Alternative methodologies (e.g., RT-PCR, digital pathology) have already been proposed for the detection of this subset of patients (Jiang et al., 2016). Among these, machine learning-based predictors showed significant results in terms of speed, accuracy, and cost-effectiveness of predicting both HER2 status and anti-HER2 treatment response (Fusco et al., 2013; La Barbera et al., 2020; Yousif et al., 2021; Farahmand et al., 2022). However, the IHC-ISH combined test remains the gold standard.

### Clinical Rationale for the Identification of HER2 Low Breast Cancer

The current evidence on HER2-targeting therapies in HER2-low breast cancer arises from several translational research studies employing various classes of monoclonal antibodies, ADC, and bispecific antibodies (Venetis et al., 2020a). Anti-HER2 vaccines and cellular immunotherapy have been also tested in HER2-low breast cancer with continuously gaining interest (Venetis et al., 2020a; Antonarelli et al., 2021a). The efficacy of trastuzumab, an anti-HER2 monoclonal antibody that binds the HER2 extracellular domain preventing receptor dimerization, is well-defined both in metastatic and early HER2+ breast cancer (Hudis 2007). The significant survival benefit obtained by the administration of this drug has been verified both in the adjuvant and neoadjuvant settings as well as in the first and subsequent lines of treatment (Slamon et al., 2001; Slamon and Pegram 2001; Piccart-Gebhart et al., 2005; Gianni et al., 2011; Slamon et al., 2011; Goldhirsch et al., 2013). Nevertheless, remarkable efficacy of trastuzumab was mainly observed in patients with HER2 3+ in IHC or 2+ and ISH amplification. In this context, the phase III study NSABP-47/NRG explored the role of adjuvant trastuzumab added to standard chemotherapy in HER2-low breast cancer (Fehrenbacher et al., 2020). However, the results of the study were negative since the invasive disease-free survival (DFS) and the overall survival (OS) were similar in the trastuzumab and the placebo arms (5-years IDFS: 89.8% with CHT plus trastuzumab *versus* 89.2% with CHT alone; hazard ratio [HR], 0.98; 95% CI, 0.76 to 1.25; *p* = 0.85; 5-years OS: 94.8% with CHT and 96.3% in CHT alone, HR, 1.33; 95% CI, 0.90 to 1.95; *p* = 0.15), thus highlighting the inefficacy of trastuzumab in HER2-low patients (Fehrenbacher et al., 2020). Pertuzumab, another anti-HER2 monoclonal antibody approved in combination with trastuzumab (dual blockade) for early and advanced breast cancer, was tested as monotherapy at two different dose levels in HER2-negative breast cancer in a phase II trial (Bachelot et al., 2019; Gianni et al., 2016; Swain et al., 2020; von Minckwitz et al., 2017). Regrettably, the results were not satisfactory in terms of efficacy, considering that only 3% (n = 2/78) of patients with IHC score 1+ and 2+ reached a partial response and 40% (n = 31/78) a stable disease (SD), with a short time to progression of 44 and 43 days in the two different dose arms (Gianni et al., 2010). The phase III SOPHIA trial demonstrated that margetuximab (a chimeric, Fc-engineered, immune-activating anti-ERBB2 immunoglobulin G1 (IgG1) monoclonal antibody sharing epitope specificity and Fc-independent antiproliferative effects with trastuzumab) in combination with chemotherapy prolongs PFS and objective response rate (ORR) if compared to trastuzumab plus chemotherapy, but the overall survival (OS) results are still awaited (Tarantino et al., 2021b; Tarantino et al., 2021c; Rugo et al., 2021). In a phase I trial of margetuximab, although HER2-low patients were included, only those with HER2+ disease experienced a response (Bang et al., 2017). Similarly, no responses were obtained in a phase II trial employing margetuximab in 22 patients with advanced breast cancer characterized by IHC score 2+ and absence of HER2 amplification, thus suggesting the efficacy of monoclonal antibodies strongly depends on high addiction to the HER2 pathway (Catenacci et al., 2020).

In HER2+ breast cancer, ado-trastuzumab-emtansine (T-DM1) is approved as adjuvant treatment for patients with residual disease after neoadjuvant therapy (von Minckwitz et al., 2019), and is still the standard of care as second-line treatment for HER2+ advanced breast cancer according to the results of TH3RESA and EMILIA trials (Diéras et al., 2017; Krop et al., 2017). Regarding T-DM1 efficacy in HER2-low breast cancer patients, the retrospective analysis of the 4,258 and 4,374 g trials showed that both PFS and ORR were significantly inferior in these patients compared to the canonical HER2+ (ORR 4.8 vs. 33.8% in the 4,258 g trial and 20 vs. 41.3% in the 4,374 g trial, while PFS 2.6 vs. 8.2 in the 4,258 g trial and 2.8 vs. 7.3 in the 4,374 g trial) (Burris et al., 2011; Krop et al., 2012). Moreover, T-DM1 was demonstrated to be less effective even in HER2 2+ and ISH-positive advanced breast cancer when compared to HER2 3+ (Yazaki et al., 2020). Trastuzumab-deruxtecan (T-DXd), another HER2-directed ADC containing a topoisomerase I inhibitor, demonstrated an impressive PFS benefit in HER2-positive pretreated breast cancer patients also in earlier lines, as emerged by the data of DESTINY-Breast 03 trial presented at ESMO 2021 congress (Cortes et al., 2021). T-DXd showed promising activity in a phase Ib trial including HER2-low patients (ORR 37%; PFS 11.1 months; mOS 29.4 months) (Modi et al., 2020). Similarly, trastuzumab-duocarmazine demonstrated interesting clinical activity in HER2-positive metastatic breast cancer patients in the phase I study (ORR 28% in HR-positive and 40% in HR-negative HER2-low metastatic breast cancer) (Banerji et al., 2019). The phase III YD985.002/TULIP trial comparing trastuzumab duocarmazine to physician’s choice treatment in patients with pre-treated HER2-positive locally advanced or metastatic breast cancer terminated enrollment (Manich et al., 2021). The results presented at the ESMO 2021 provide evidence that treatment with SYD985 may represent a new therapeutic option for these patients. PF-06804103 is the other anti-HER2 ADC that was tested in HER2-low breast cancer patients in a phase I trial. To date, no results from this trial have been published, except for an abstract, that does not separately analyze the HER2-low cohort (Meric-Bernstam et al., 2019). Among bispecific antibodies, zenocutuzumab is an anti-HER2 and HER3 IgG1 more potent than pertuzumab in inhibiting HER2-HER3 heterodimerization which was initially tested in hormone receptor (HR)+/HER2-low breast cancer xenograft models, suggesting a potential synergistic effect with endocrine therapy (Geuijen et al., 2018; Antonarelli et al., 2021b). The drug was successively tested in clinical trials, among which a phase II, single-arm study enrolling endocrine-resistant metastatic breast cancer patients after progression to a CDK4/6 inhibitor (Pistilli et al., 2020; Eiger et al., 2021). Among the 50 patients enrolled, the clinical benefit rate at 24 weeks was 16.7%, with one patient that obtained a PR (Pistilli et al., 2020). Several clinical studies are currently ongoing with T-Dxd and other ADCs including HER2-low breast cancer patients **(** Table 1).

**TABLE 1:** Ongoing and recently completed clinical trials using HER2-targeting agents in HER2-low and/or “ultra-low” breast cancer patients.

NCT	Class of drug	Anti-HER2 agent	Phase	Cohort (timeline)	No. of patients	HER2 status	Primary endpoint	Results
NCT01828021	Monoclonal antibody	Margetuximab	II	2013–2020	25	IHC 2+ or 1+, ISH-negative, score ≥10.5 by HERmark testing	OR	Pending
NCT02491892		Pertuzumab	II	2015	79	IHC 1+ or 2+, ISH negative	ORR	ORR, 2.5%
NCT01275677		CHT ± trastuzumab	III	2011–2021	3270	IHC 1+ or 2+ ISH-negative	IDFS	5-years IDFS: 89.8% with CHT plus trastuzumab v 89.2% with CHT. HR: 0.98; 95% CI, 0.76 to 1.25; *p* = 0.85
NCT02564900	ADC	T-DXd	I	2015–2022	292	HER2-low (unspecified)	ORR	Pending
NCT02277717		SYD985	I	2014–2020	185	IHC 1+ or 2+ ISH-negative	Dose-limiting toxicity	ORR, 28% in HR+; ORR, 40% in TNBC
NCT04602117		SYD985+ weekly paclitaxel	I	2021–2022	27	HER2-low (unspecified)	AE, CBR, ORR	Ongoing
NCT03523572		T-DXd+ Nivolumab	I	2018–2021	99	IHC 1+ or 2+ ISH-negative	Dose-limiting toxicity, ORR	Pending
NCT03368196		T-DXd	I	2017–2021	12	Any HER2 expression (IHC 1–3 and/or ISH positive	AE	Pending
NCT04556773		T-DXd+ ICI/CHT/ET	I	2020–2023	185	IHC 1+ or 2+ ISH-negative	AE	Ongoing
NCT03734029		T-DXd	III	2018–2023	557	IHC 1+ or 2+ ISH-negative	PFS	Pending
NCT04042701		T-DXd+ Pembrolizumab	I	2019–2021	115	IHC 1+ or 2+ ISH-negative	DLTs, ORR	Pending
NCT04494425 (Destiny Breast 06)		T-DXd	III	2020–2025	850	IHC 1+ and IHC 2+/ISH-negative or IHC >0 < 1+	PFS	Ongoing
NCT04420598 (DEBBRAH)		T-DXd	II	2020–2023	39	IHC 1+ or 2+ ISH-negative	CNS ORR	Ongoing
NCT04553770		T-DXd ± anastrozole	II	2020–2025	88	IHC 1+ and IHC 2+/ISH-negative	pCR	Ongoing
NCT04742153		MRG002	II	2021–2022	66	HER2-low (unspecified)	ORR	Ongoing
NCT02512237		ARX788	I	2015–2020	9	IHC 2+/ISH-negative	Maximum tolerated dose	Pending for HER2-low cohort
NCT03255070		ARX788	I	2017–2021	190	IHC 2+/ISH-negative	TEAE, ORR	Pending
NCT05018676		ARX788	II	2021–2023	54	IHC 1+ or 2+ ISH-negative	ORR	Ongoing
NCT03052634		RC48-ADC	I/II	2016–2021	121	IHC 1+ or 2+ ISH-negative	RP2D	Pending
NCT04400695		RC48-ADC	III	2020–2023	366	IHC 1+ or 2+ ISH-negative	PFS	Ongoing
NCT03602079		A166	I/II	2018–2021	82	HER2 expression ≥1 or ISH+	Maximum Tolerated Dose, ORR	Ongoing
NCT03284723		PF-06804103	I	2017–2021	106	IHC 2+ without ISH confirmation	DLTs AE, DR, PFS, TTP	Pending
NCT04147819		BAY2701439	I	2020–2027	213	HER2-expressing tumors (including HER2 3+, 2+ and 1+)	TEAE, TESAE, DLT, ORR	Ongoing
NCT03944499		FS-1502	I	2019–2021	92	IHC 1+ or 2+ ISH-negative	DLT, MTD, RP2D, ORR	Pending
NCT02952729		XMT-1522	I	2016–2021	120	IHC 1+ or 2+ ISH-negative	Maximum Tolerated Dose	Pending
NCT01355393	Vaccine	HER-2/neu peptide	I/II	2011–2020	50	IHC 1+, 2+ or 3+ or ISH positive	MBD	Pending
NCT01730118		AdHER2/neu DC	I	2012–2021	33	IHC 1+, 2+ or 3+ or ISH positive	AE, ORR	Pending
NCT01479244		Nelipepimut–S	III	2011–2016	758	IHC 1+ or 2+ ISH-negative	DFS	3-years DFS 77.1% with NP-S group v 77.5% with placebo
NCT01570036		Nelipepimut–S+ trastuzumab	II	2012–2020	275	IHC 1+ or 2+ ISH-negative	DFS	24-months DFS, 88% in SG v 82% in CG
NCT00524277		GP2/AE37	II	2007–2020	456	IHC 1+, 2+, 3+ and/or FISH ratio >1.2	Disease recurrence	5-years DFS, 88% SG v 81% CG; 55-months DFS, 89% SG v 51% CG
NCT03321981	Bi/trispecific antibody	MCLA-128+ endocrine treatment	II	2017–2021	101	IHC 1+ or 2+	CBR	Pending
NCT02892123		Zanidatamab	I	2016–2021	418	IHC 1+, 2+ or 3+	DLTs, AE	Pending
NCT03448042		BTRC4017A	I	2018–2021	449	No specific criteria provided	AE	Pending
NCT04162327		IBI315	I	2019	191	No specific criteria provided	AUC, Cmax, T1/2, Vd	Pending
NCT00522457		Ertumaxomab	II	2007–2011	19	IHC 1+ or 2+ ISH-negative	ORR	ORR, 0%; DCR, 53,8%
NCT00351858		Ertumaxomab	II	2006–2009	40	IHC 1+ or 2+ ISH-negative	Clinical efficacy (unspecified)	Not provided
NCT05013554		SAR443216	I	2021–2025	184	HER2-low (unspecified)	MTD, ORR, TEAE, DLT	Ongoing
NCT02829372		GBR1302	I	2016–2020	36	IHC 2+, ISH negative	MTD, DLT, AE	ORR, 0%, DCR, 10%
NCT00900627	Tyrosine kinase inhibitor	Paclitaxel ± AZD8931	I/II	2009–2015	330	HER2-low (unspecified)	PFS	No differences in PFS observed with AZD8931+ Paclitaxel vs. Paclitaxel alone (median PFS, 8.7 vs. 9.1 months, *p* = 0.679)

NCT, number of clinical trial; IHC, immunohistochemistry; ISH, *in situ* hybridization; ORR, Objective response rate; ADC, Antibody-drug conjugates; DCR, Disease control rate; OR, overall response; DoR, Duration of response; TTR, Time to response; PFS, Progression-free survival; OS, Overall survival; PK, Pharmacokinetic analysis; AE, Adverse events; AUC, Area under the plasma concentration versus time curve; TEAE, Treatment emergent adverse events; Cmax, Maximum concentration; DFS, Disease-free survival; BCFS, Breast cancer-free survival; RFI, Recurrence-Free interval; DRFI, Free From Distant Recurrence interval; Tmax, Time to reach maximum observed concentration; pCR, pathological complete response; PR, Partial response; MRT, Mean residence time; CBR, Clinical benefit rate; ADA, Anti-drug antibody; T1/2, Terminal elimination half-life; Vd, Volume of distribution; RO, Receptor occupancy; Nab, Neutralizing antibody; ICI, Immune checkpoint inhibitor; CHT, chemotherapy; ET, endocrine therapy; RP2D, recommended phase II dose; CNS, central nervous system; TESAE, treatment-emergent serious adverse event; DLT, dose-limiting toxicity.

Cancer vaccines have reached clinical trials based on the preclinical evidence of a synergistic effect with trastuzumab in HER2-low breast cancer (Tarantino et al., 2020; Corti et al., 2021a). Regrettably, the results of nelipepimut (an E75 HER2 peptide vaccine) plus trastuzumab versus trastuzumab plus placebo were negative in the adjuvant setting of HER2-low breast cancer (disease-free survival (DFS) was similar in the two groups of treatment: HR, 0.62; 95% CI 0.31–1.25; *p* = 0.18) (Clifton et al., 2020). Nevertheless, in patients with TNBC, DFS was improved in the experimental arm versus control (HR, 0.26; 95% CI, 0.08–0.81, *p* = 0.01) (Clifton et al., 2020). Similar results were obtained in two phase II clinical trials in the adjuvant setting with the other two HER2 peptide vaccines, GP2 and AE37 (Brown et al., 2020). With AE37, a DFS benefit was noted in an advanced stage, HER2 under-expression, and TNBC, while with GP2 there were no recurrences in patients with HER2-positive disease (Brown et al., 2020). A recent meta-analysis including 24 studies with E75 and GP2 vaccines showed a DFS benefit with the E75 vaccine (You et al., 2021).

### HER2 “Ultra-Low”: A New Entity in the Field?

According to the findings of retrospective analyses of the National Surgical Adjuvant Breast and Bowel Project (NSABP) B-31 trial and North Central Cancer Treatment Group (NCCTG) N9831 trial, a subset of breast cancer patients resulted negative for HER2 biomarker assessment by ISH/IHC, benefited from anti-HER2 therapy (MacNeil et al., 2020). This postulates that the current HER2 assessment does not fully associate with HER2 signaling dysfunction. It should be noted, however, that a subset of HER2-negative tumors tested in core biopsy samples might respond to targeted therapy due to intra-tumor heterogeneity phenomena (Ercoli et al., 2017). Moreover, HER2 targeting may theoretically be possible even in score 0 tumors showing staining, albeit incomplete and faint, in ≤10% of tumor cells. To identify HER2-negative tumors susceptible to HER2 inhibition, several studies investigated the existence of a predictive marker that could detect patients benefiting from this therapy or the use of a functional signal profiling test to identify abnormal HER2-driven signaling activity.

### Insights From the Biological Landscape of HER2 Low Expression

Pathologists and oncologists are currently putting efforts into defining all possible HER2 entities for achieving the most accurate stratification of the patients and the selection of an appropriate therapeutic approach. In this regard, remarkable endeavors aimed to deepen into the clinical and molecular landscape of HER2-low breast cancer (Dieci and Miglietta 2021). The recent study published by Carsten Denkert et al. sought to determine this novel breast cancer subgroup by comparing its clinical and molecular characteristics with those of HER2-zero (i.e., complete HER2-negativity determined by IHC) breast cancer (Denkert et al., 2021). To achieve this, the authors performed IHC analysis in a cohort of 2,310 patients with HER2 non-amplified primary breast cancer treated with neoadjuvant combination chemotherapy in four prospective clinical trials. In terms of HR status, a significant difference was observed between the two groups since HER2-low breast cancer was enriched with HR+ tumors, while the HER2-zero cohort was enriched with HR- cases. Such correlation between HR signaling and HER2-low expression has also been reported in a retrospective, large-cohort, gene expression profiling study, where PAM50-based-subtypes accounted for approximately 80% of HER2-low-positive cases (Miglietta et al., 2021). Concerning the pathological complete response (pCR) data, in the HR+ cohort, the authors observed a lower pCR in patients with HER2-low breast cancer compared to those with HER2-zero disease with no effect on long-term survival (Denkert et al., 2021). On the contrary, even though such an association between HER2 status and pCR was not found in the TNBC group, longer disease-free and overall survival were reported in HER2-low patients in comparison with HER2-zero patients. Albeit this result was specifically seen in patients who did not achieve a pCR after neoadjuvant chemotherapy, to date, the prognostic role of HER2-low and potential correlation with sensitivity to chemotherapy still remain matters of controversy (Tarantino et al., 2020; Mutai et al., 2021; Schettini et al., 2021). In addition to these intriguing findings, the study published by Schettini et al. pointed out another important difference between these two entities which is related to gene expression data (Schettini et al., 2021). Indeed, it has been demonstrated that *ERBB2* and luminal-related genes had higher levels of expression in HER2-low compared to HER2-cases in the HR+ group. Although the abovementioned studies highlighted major diversities between HER2-low and HER2-zero breast cancer in terms of biological and molecular characteristics, no robust evidence is available on whether HER2-low should be considered as a clinically separate entity (Dieci and Miglietta 2021; Omar and Arafat 2021). Considering that most of the available evidence derives from retrospective studies, results from the currently ongoing clinical trials are eagerly awaited.

### HER2 Targeting in HER2 Score 0 and/or Mutant Breast Cancer

Although targeting HER2 in HER2-negative breast cancer could sound like an oxymoron, it has been demonstrated to be at least theoretically possible. Hence, among IHC score 0 tumors, a substantial proportion of cases shows incomplete and faint staining in ≤10% of tumor cells. These HER2 “ultra-low” breast cancers might explain the positive results in some studies targeting HER2 in HER2-negative tumors. For example, a study by Bose et al. published in 2013 showed that HER2 pathogenic activating mutations occur irrespectively of HER2 IHC status as they do not necessarily lead to protein overexpression, representing an alternative mechanism for activating the HER2 pathway in breast cancer (Bose et al., 2013). Out of the eight HER2-mutated samples without gene amplification, for whom IHC data are available in the article, one was HER2-zero and harbored a V777L ERBB2 mutation, demonstrated to be an activating mutation as it strongly increased the phosphorylation of signaling proteins, indicating enhanced activity of the tyrosine kinase (Bose et al., 2013). Regrettably, the presence of HER2 positive cells within the score 0 category was not annotated in this study. Moreover, HER2 V777L-mutated breast cancer cell lines showed sensitivity to TKIs: lapatinib and neratinib, thus suggesting a possible role of HER2 targeting also in particular cases of HER2 “ultra-low” breast cancer (Bose et al., 2013). Regarding clinical studies, a phase II trial explored the efficacy of neratinib, a pan-HER inhibitor, in HER2-mutant advanced breast cancer, including 14 non-amplified breast cancers (Exman et al., 2019). Five of them (i.e., 36%, 90% CI 15–61%) obtained clinical benefit, including one complete response (CR), 1 PR, and 3 SD ≥ 6 months, while median PFS was 5 months (90% CI 2–8) (Exman et al., 2019). Nevertheless, this evidence is based on very rare findings given that HER2 activating mutations are described to occur in less than 2% of breast cancer, with a higher frequency in HR-positive compared to TNBC and in lobular than in ductal histology (Exman et al., 2019).

### Mismatch Repair-Deficient ER+ Breast Cancers With MutL Loss

A recent study has proposed the application of anti-HER agents for ER+/HER2- breast cancers in the context of mismatch repair (MMR) status (Punturi et al., 2021). MMR is known as one of the fundamental DNA repair pathways (Piciotti et al., 2021) Defects in the MMR system are commonly due to molecular alterations involving the MutS and MutL dimeric homologs (Corti et al., 2019; Lopez et al., 2020). These complexes interact with each other to regulate the recognition and cleavage of incorrect base insertions (Sajjadi et al., 2021a). Almost 15–17% of ER+/HER2− breast cancer patients are correlated with endocrine treatment resistance due to MutL deficiency (Haricharan et al., 2017; Sajjadi et al., 2021). Punturi et al. have shown that in endocrine-treated, ER+/HER2− patients, the loss of MutL expression could activate HER2 by protecting it from lysosomal protein trafficking. Owing to this activation, MutL loss has been proposed as a marker to stratify ER+/HER2− breast cancer patients who would respond to anti-HER agents. This observation was based on multiple experimental model systems. Accordingly, based on gene expression microarray data from independent datasets, it has been shown that ER+/HER2− tumors with MutL loss have a relatively higher expression of ERBB2 compared to those with MutL proficient tumors, but this was not observed in the absence of treatment. These results were not only observed in cell line models but also in patient-derived xenograft models where ER^+^/HER2^−^ tumors with MutL loss showed an increase in membrane HER2 levels after fulvestrant treatment. This finding was reflected by an increased sensitivity to HER inhibition. The data published in this study suggest that MutL loss inclines ER+/HER2− tumors to respond to a combination of anti-HER agents and endocrine treatment (Punturi et al., 2021). On the other hand, the currently available HER2 tests (IHC, ISH) provide information on the protein expression or gene amplification, which do not include the functional status of the HER2 biomarker. Therefore, the measurement of the signaling pathway activity of HER2 in addition to the common methods could stratify HER2-negative patients eligible for HER2 targeted therapies (Huang et al., 2017). In this approach, patients’ live tumor cells are applied on a biosensor that can identify dynamic HER2-driven signaling dysfunction. Among the HER2-negative samples, almost a quarter (27 out of 114 patients, 23.7%) have been related to abnormal HER2 signaling (MacNeil et al., 2020). This test has demonstrated the efficacy of various HER2 signal inhibitors in HER2-negative breast cancers with abnormal HER2 signaling (MacNeil et al., 2020). Further studies and clinical trials to evaluate the efficacy of HER2-targeted therapy in such patient populations are warranted.

## Discussion

**FIGURE 2:**
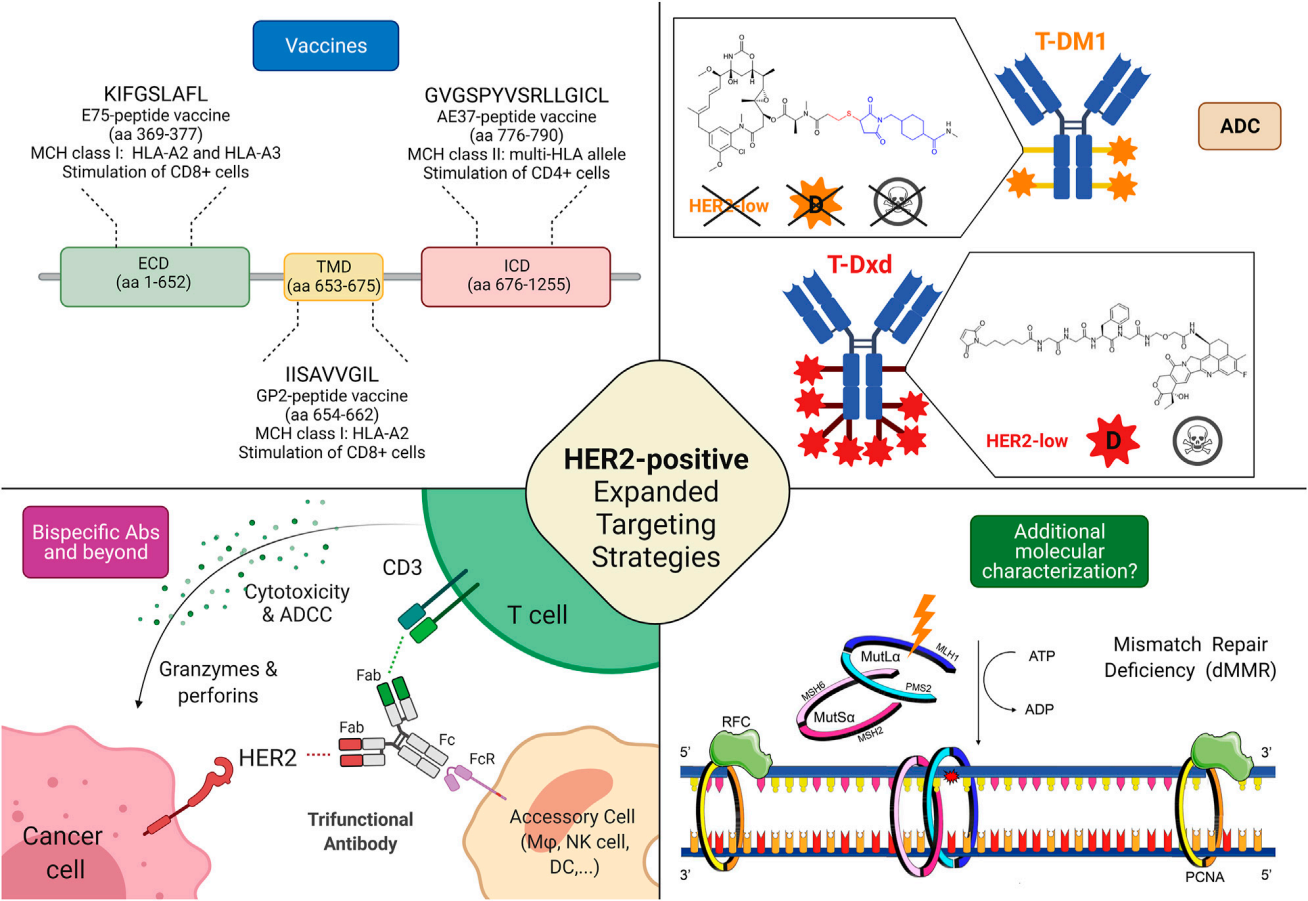
Possible targeting strategies in HER2-low and HER2-negative breast cancers. **1) Cancer vaccines:** most of the research efforts related to cancer vaccines against HER2 regard targeting HER2-related peptides, such as E75 (ECD), GP2 (TMD), and AE37 (ICD); Interestingly, while a phase II trial testing AE37-based vaccine in the advanced stage (in the study defined as stage IIB or greater) did not demonstrate statistically significant survival benefit (5-years DFS) in the overall population, it showed a 5-years DFS of 83% in subjects with HER2-low disease, compared with 62.5% in the GM-CSF-only arm (HR, 0.375; CI: 0.142–0.988; *p* = 0.039). Similarly, in a subgroup with stage IIB or greater TNBC, a trend toward improved DFS with vaccination was found. The growing body of evidence coupled with subset analyses of this phase II trial led to another phase II clinical trial investigating AE37 in combination with pembrolizumab (NSABP FB-14) in TNBC patients with metastatic disease (NCT04024800). **2) Antibody-drug conjugates:** several trials are currently testing ADC in HER2-low disease, with interesting results, particularly regarding those equipped with diffusable cytotoxic moiety as well as cleavable linkers, possibly accounting for bystander killing effect (skull symbol); **3) Bispecific antibodies** (bsAbs) and trifunctional antibodies are characterized by the ability to target (at least) two different epitopes, enabling either inhibition of multiple oncogenic pathways or the forced connection between immune cells and cancer tissue. Several anti-HER2 bsAbs are under development, although only a minority is specifically investigated in HER2-low disease; **4) Additional molecular characterization** is a possible new prospective, in the light of recent findings describing the predictive value of mismatch repair deficiency related to response to HER2 blockade in HER2-negative breast cancer. Abbreviations: T-DM1, trastuzumab emtansine; T-DXd, trastuzumab deruxtecan; HER2, human epidermal growth factor receptor 2; ECD, extracellular domain; TMD, transmembrane domain; ICD, intracellular domain; aa, amino acid; MHC, major histocompatibility complex; HLA, human leukocyte antigen; ADC, antibody-drug conjugate; D, diffusible cytotoxic moiety; MSH6, MutS Homolog 6; PMS2, PMS1 Homolog 2; MSH2, MutS Homolog 2; MLH1, MutL homolog 1; DFS, disease-free survival; bsAbs, bispecific antibody; CD, cluster of differentiation; ATP, Adenosine triphosphate; ADP, adenosine diphosphate; Fab, antigen-binding fragment; Fc, fragment crystallizable; FcR, Fc receptor; dMMR, mismatch repair deficient; ADCC, Antibody-dependent cellular cytotoxicity; RFC, replication factor C; PCNA, Proliferating cell nuclear antigen. Created with biorender.com.

Globally, HER2 expression is being increasingly perceived as a continuum spectrum, going beyond the classical dichotomous distinction between HER2-positive and HER2-negative cancer that led the treatment choice until today, especially in breast cancer. For this reason, it is becoming crucial to collect more solid evidence on targeting HER2 based on the whole spectrum of HER2 expression. Hence, a widely different efficacy of different classes of drugs and even of different drugs within the same class has been highlighted in the clinical trials according to the HER2 status (Figure 2). Monoclonal antibodies globally demonstrated to be ineffective in HER2-low breast cancer, because their activity relies mainly on the blockade of aberrant HER2 signaling via dimerization inhibition, HER2 internalization, and antibody-dependent cellular cytotoxicity (Hudis 2007; Eiger et al., 2021). They bind to the extracellular domain of the receptor thus they are more effective when the receptor is overexpressed, allowing more drugs to bind on the cell membrane inducing antibody-dependent cellular cytotoxicity. Moreover, monoclonal antibodies intrinsically act on the cell on which they bind, so in case of high intratumoral heterogeneity, their efficacy is impaired (Tarantino et al., 2020).

On the other hand, ADCs can overcome some of the limitations encountered by monoclonal antibodies vehiculating and releasing a cytotoxic payload that can be internalized also by the surrounding cells that do not express HER2 (bystander effect) (Tarantino et al., 2021a; Corti et al., 2021b). In this perspective, the characteristic of the ADC, mainly the cytotoxic activity of the payload, the drug-antibody ratio (DAR), and the cleavability of the linker through which the payload is charged on the antibody, make the difference. T-DM1 is composed of trastuzumab and DM1 (emtansine), a tubulin polymerization inhibitor, linked through an uncleavable linker with a DAR of 3.5 (Krop et al., 2010). T-DM1 needs to be internalized and the antibody degraded before executing its cytotoxicity (Skeie et al., 2020). The mandatory internalization prevents the possibility of targeting HER2 non-expressing surrounding cells. In T-Dxd, on the contrary, trastuzumab is conjugated with deruxtecan, a topoisomerase I inhibitor with a DAR of eight *via* a cleavable but stable linker (Doi et al., 2017). The theoretical advantage given by the payload more potent, with higher DAR and by the cleavable linker thanks to the lysosomal enzymes present both in the endosomes and in the microenvironment translates in better activity in clinical trials enrolling HER2-low breast cancer patients, as previously reported. The presence of enzymes capable of cleaving the linker thus releasing the chemotherapeutic agent in the extracellular space and the higher payload confer to T-Dxd activity also on the cells that express HER2 at a lower level or even that do not express it at all (Tarantino et al., 2020). In Trastuzumab-duocarmazine the payload is a potent alkylating agent with a DAR of 2.8 and a cleavable linker (Banerji et al., 2019). On the whole, ADCs permit the target of HER2 independently from the cell and even the tumor addiction to HER2 pathway provided that a cleavable linker and a potent chemotherapeutic agent seem to be necessary to exert a sufficient bystander effect. Based on these findings, as T-Dxd and trastuzumab-duocarmazine demonstrated promising activity in HER2-low breast cancer but no data are currently available on survival endpoints, HER2-low expression in breast cancer can be classified as a tier IIb according to ESMO Scale for Clinical Actionability of molecular Targets (ESCAT), hoping that this evidence could reach a tier I in the future when more solid data on survival endpoints will be available (Mateo et al., 2018; Crimini et al., 2021). Focusing on HER2-negative breast cancer, the most promising therapeutical strategies are probably HER2 vaccines, as they assume that breast cancer cells express HER2 at a higher level than healthy tissues, even in cases of HER2-low and ultra-low breast cancers by IHC (You et al., 2021). In this perspective, the previously cited metanalysis supports further clinical trials on the topic to assess which subgroups of patients benefit from this approach (You et al., 2021). Moreover, the clinical validation of HER2 targeting in MutL deficient breast cancer is required to assess if this strategy could be implemented in the future.

In conclusion, targeting HER2 is revealing a fine and increasingly more complex work concerning the growing knowledge of the topic, that determines a deeper understanding of molecular mechanisms underlying the variable expression of HER2 in different tumors and even in different cells of the same tumor, a knowledge that is at the same time necessary to develop more effective therapies for breast cancer patients. For this, a tailored approach is warranted to assess HER2 status. Further prospective studies addressing the role of HER2 ultra-low expression along with additional complementary biomarkers would bring us a further step closer to the realization of the potentials of precision medicine for these patients.
